# ATM Expression and Activation in Ataxia Telangiectasia Patients with and without Class Switch Recombination Defects

**DOI:** 10.1007/s10875-025-01857-3

**Published:** 2025-01-24

**Authors:** Fereshte Salami, Tannaz Moeini Shad, Nazanin Fathi, Hanieh Mojtahedi, Marzie Esmaeili, Sepideh Shahkarami, Ladan Gol Mohammad Pour Afrakoti, Parisa Amirifar, Samaneh Delavari, Hassan Nosrati, Azadehsadat Razavi, Mohammad Reza Ranjouri, Mahsa Yousefpour, Zahra Hamidi Esfahani, Gholamreza Azizi, Mahmoudreza Ashrafi, Nima Rezaei, Reza Yazdani, Hassan Abolhassani

**Affiliations:** 1 Research Center for Immunodeficiencies, Pediatrics Center of Excellence, Children´s Medical Center, Tehran University of Medical Sciences, 62 Qarib St., Keshavarz Blvd, Tehran, 14194 Iran; 2 Primary Immunodeficiency Diseases Network (PIDNet), Universal Scientific Education and Research Network (USERN), Tehran, Iran; 3 Department of Pediatrics, Dr. von Hauner Children’s Hospital, University Hospital, Ludwig-Maximilians- Universität München (LMU), Munich, Germany; 4 Medical Genetics Network (Megene), Universal Scientific Education and Research Network (USERN), Tehran, Iran; 5 Department of Immunology, School of Medicine, Tehran University of Medical Sciences, Tehran, Iran; 6 Department of Radiotherapy Oncology, Cancer Research Center, Tehran University of Medical Sciences, Tehran, Iran; 7 Department of Neurology, Thomas Jefferson University, Philadelphia, PA USA; 8 Non-communicable Diseases Research Center, Alborz University of Medical Sciences, Karaj, Iran; 9 Department of Pediatric Neurology, Children’s Medical Center, Tehran University of Medical Sciences, Tehran, Iran; 10 Division of Immunology, Department of Medical Biochemistry and Biophysics, Biomedicum, Karolinska Institute, Solnavägen 9, floor 9D, Stockholm, 17165 Sweden

**Keywords:** Inborn errors of immunity, primary immunodeficiency, ataxia telangiectasia (A-T), Class switch recombination (CSR), Phosphorylated ATM (p-ATM), Ionizing radiation, Severe and mild, Immunodeficiency

## Abstract

**Background:**

Ataxia telangiectasia mutated (ATM) kinase plays a critical role in DNA double-strand break (DSB) repair. Ataxia telangiectasia (A-T) patients exhibit abnormalities in immunoglobulin isotype expression and class switch recombination (CSR). This study investigates the role of residual ATM kinase expression and activity in the severity of A-T disease.

**Methods:**

A-T patients with defined genetic diagnoses were classified based on CSR and based on the severity of their medical complications. Isolated peripheral blood mononuclear cells from any patient were evaluated before and after exposure to 0.5 Gy ionizing radiation for one minute. Western blotting was performed to identify the expression of ATM and phosphorylated ATM (p-ATM) proteins compared to age-sex-matched healthy controls.

**Results:**

In severe A-T patients (*n* = 6), the majority (66.7%) had frameshift mutations, while 33.3% had nonsense mutations in the *ATM* gene. The mild group (*n* = 3) had two cases of splice errors and one missense mutation. All patients with CSR defect had elevated IgM serum levels, whereas all switched immunoglobulins were reduced in them. Expression of ATM and p-ATM proteins was significantly lower (*p* = 0.01) in all patients compared to healthy controls, both pre-and post- and post-radiation. Additionally, low ATM and p-ATM protein expression levels were linked with the clinical severity of patients but were not correlated with CSR defects.

**Conclusion:**

Expression and activation of ATM protein were defective in A-T patients compared to healthy controls. Altered expression of ATM and p-ATM proteins may have potential clinical implications for prognostic evaluation and symptom severity assessment in individuals with A-T.

**Supplementary Information:**

The online version contains supplementary material available at 10.1007/s10875-025-01857-3.

## Introduction

Ataxia telangiectasia (A-T) is a rare neurodegenerative syndrome caused by variations in the ataxia-telangiectasia mutant (ATM) protein [1]. It is inherited in an autosomal recessive pattern [2]. Approximately 40 years ago, A-T was identified as a distinct disease [3]. Its incidence in the world is estimated to be 1 in 40,000, with 1 in 100,000 live births in the United States [4]. While A-T can affect people of all races, it is more common in ethnic groups with a high prevalence of consanguinity, such as Iran and other Middle Eastern countries [5]. The ATM plays a crucial role in regulating and monitoring the double-stranded DNA breaks (DSBs) [6]. It also participates in the repair of DSBs, which could explain the heightened radiosensitivity and susceptibility to cancer in A-T cells [7, 8]. ATM is a serine/threonine protein kinase involved in numerous pathways, including oxidative stress, cell cycle checkpoints, gene transcription and expression, apoptosis, and more [9, 10]. A-T is a condition characterized by a gradual onset of cerebellar ataxia, oculocutaneous telangiectasia, inborn errors of immunity, and chromosomal instability leading particularly to leukemia and lymphoma in children [4, 11]. Additionally, non-hematologic tumors such as those in the stomach, breast, ovary, liver, uterus, and melanoma have been observed in A-T patients [12].

Immune deficiency is a concern in about one-third of cases, which can make patients more susceptible to respiratory infections and other illnesses [13]. Particularly, in A-T patients, a class switch recombination (CSR) mechanism in B cells is defective, and therefore, the production of diverse antibody isotypes in response to antigen stimulation during primary B cell maturation might be impaired [14]. During the process of CSR, the constant region of the heavy chain changes, while the variable domain remains unaltered from the original immunoglobulin (Ig) [15]. This process involves the activation of DNA damage sensors, including ATM, which harmonize downstream pathways in mainly cell-cycle checkpoints and later apoptosis and senescence [16].

At present, there is no definitive treatment strategy for A-T, but some treatments may help alleviate immune system symptoms/infections, prevent cancer development, and slow down neurodegeneration [17–20]. Studies have shown that patients with severe immune defects exhibit defective CSR, as evidenced by an increase in IgM levels and a decrease in IgA, IgE, and IgG levels [21], the relationship between CSR mechanism defects, ATM molecule expression, and kinase activity remains unclear. ATM protein expression and kinase activity could potentially contribute to the heterogeneity of the disease. Moreover, identifying the underlying genetic mutation impact via investigation of the residual ATM protein expression and function could improve prognosis estimation and treatment efficacy. In this study, we aimed to evaluate the expression of ATM protein and its kinase activity in A-T patients with CSR defect (CSR-D) and CSR normal (CSR-N) conditions to reveal the expression of residual ATM kinase and its association with the severity of the disease and the phenotypic-genotypic correlation in A-T patients.

## Materials and Methods

### Study Population

In this study, peripheral blood samples were collected from available and genetically defined A-T patients recruited by the Research Center for Immunodeficiencies (RCID), which is the Iranian Immunodeficiency Registry Center at Children’s Medical Center Hospital in Tehran, Iran in 2022 [22]. Individuals diagnosed with A-T were selected based on European Society for Immunodeficiency (ESID) guidelines [23], including clinical presentation of ataxia and at least two of the following: oculocutaneous telangiectasia, elevated alpha-fetoprotein (AFP), lymphocyte A-T karyotype with translocation chromosome 7:14, and cerebellar hypoplasia on magnetic resonance imaging (MRI). In all studied A-T patients, *ATM* mutations were identified primarily by genomic sequencing [24, 25]. CSR proficiency (CSR-D and CSR-N) were assigned as determined in Amirifiar et al. To differentiate between the two groups, they used nested PCR to increase the level of Sµ–Sα fragments and stimulated peripheral blood mononuclear cells (PBMCs) using sCD40L + rIL-4 to promote B-cell proliferation. This allowed them to evaluate the levels of IgA and IgE in CSR, instead of measuring IgE secretion in the supernatant from human B cells as done in previous studies [26]. Also, based on the medical complications, patients were divided into two different groups: a severe group and a mild group [25]. Additionally, samples from age-sex-matched healthy individuals were considered as negative controls. All participants were alive and treated based on the Middle East and North Africa management guidelines for inborn errors of immunity [27]. All the candidates and or their legal guardians accepted informed consent, and the study protocol was reviewed by the Tehran University of Medical Sciences Ethics Committee (IR.TUMS.CHMC.REC.1398.030).

### Immunoblotting for ATM Expression and ATM Activity Assays

As we hypothesized that the residual expression of ATM kinase might lead to mild and CSR-normal A-T patients, we determined whether the patient’s cells showed ATM kinase expression and activity as assessed by the detection of phosphorylated substrates of ATM using phospho-specific antibodies. For ATM protein expression assays, a total number of lymphocytes of peripheral blood samples was counted, and peripheral blood mononuclear cells were extracted and resuspended in phosphate-buffered saline (PBS) buffer and then lysed on ice by Radioimmunoprecipitation assay (RIPA) buffer (Santa Cruz). For ATM activity assays, the derived cells of each patient were divided into two parts. The first 1 × 10^6^ cells were exposed to 0.5 Gy ionizing radiation and harvested after one minute before the cell pellets were resuspended in PBS buffer and lysed on ice by sonication, and 1 × 10^6^ cells, as non-activated ATM sample, remained without radiation [28, 29]. Ten microgram protein/samples from irradiated and non-irradiated whole-cell lysates of A-T patients and the control group were loaded on 10% sodium dodecyl-sulfate polyacrylamide gel electrophoresis (SDS-PAGE), and then the proteins were transferred to polyvinylidene difluoride (PVDF) membrane (GE Healthcare). PVDF strips were subjected to immunoblotting, and the protein bands were visualized using the enhanced chemiluminescence (ECL) system (Amersham, Little Chalfont). Antibodies used for immunoblotting were ATM #PLA0086 (Sigma-Aldrich), phosphorylated ATM Ser1981 #AF1655 (p-ATM, R&D Systems, Abingdon), β-actin housekeeping #8457 (Cell Signaling Technology). Subsequently, Anti-rabbit IgG, HRP-linked Antibody #7074S (Cell Signaling) was applied. Both patient and control samples were exposed simultaneously. The proteins were visualized by using the ChemiDoc Imaging System (Biorad). To ensure consistency, all signals were normalized using β-actin as a reference using ImageJ Software (NIH).

### Statistical Analysis

All the provided data are presented by the median (interquartile range, IQR) unless stated otherwise. We used a non-parametric test to compare distributed quantitative data. Specifically, we employed two related sample tests, namely the Mann-Whitney U test and the Wilcoxon sign test, for the analysis of demographic data. Furthermore, chi-square was used for the analysis of qualitative data. All statistical analysis was performed by SPSS (IBM SPSS Statistics for Windows, version 22.0.). The statistical significance level was at *p*-value < 0.05. Both the mean (standard deviation, SD) and median (interquartile range, IQR) are presented due to the Kolmogorov-Smirnov test for normality. Although we determined that the data did not follow a normal distribution, we included both measures (mean ± SD and IQR) for all groups tested to provide a comprehensive representation of the data.

## Results

A total of nine A-T cases, including six females and three males with a median (IQR) age of 9.0 (7.0–11.0) years at the time of the study, were consented to the present study. The frequency of parental consanguinity was more than 70% in patients. Four A-T patients were classified as CSR-D, and they were all females with hyper IgM immunologic profiles; three of these four patients belonged to the group of clinically severe A-T patients. The major demographic and immunological findings of the patients are provided in Table 1.

**Table 1:** Demographic and immunologic features of A-T patients (*N* = 9)

Parameter		All patients (*N* = 9)	Patients with severe form (*N* = 6)	Patients with mild form (*N* = 3)	Controls (*N* = 3)	*p*. value severe vs. mild	patients with CSR defect (*N* = 4)	patients with CSR normal (*N* = 5)	*p*. value	
Age at the study time, years; median (IQR)		9 (7–11)	9.5(7.75-12)	7(7–12)	31 (29–35)	0.35	11(7.75-12)	9(5.5–9.5)	0.13	
Age at diagnosis, years; median (IQR)		3(1.75–7.5)	3.5(1.74-8)	3(1.5- 7)	-	0.79	4(1.5–7.25)	2(1.75–7.5)	0.90	
Age at onset of disease; years median (IQR)		2(1.5–3.5)	1.75(1.37–2.5)	3(1.5-4)	-	0.35	2.25(1.12–3.75)	2(1.5-3)	0.90	
Age at onset Ataxia, years; median (IQR)		1.5(1.25-3)	1.75(1.37–2.5)	1.5 (1–4)	-	0.79	1.5(1.12–3.37)	2(1.25-3)	0.61	
Age at onset Telangiectasia, years; median (IQR)		3.5(2–4)	2(2–4)	4(3–4)	-	0.22	3.5(2.25–4.75)	2(2-)	0.35	
Female; number (%)		6(66.7)	4(66.7)	2(66.7)	2(66.7)	1.0	4(100)	4(80)	0.06	
Parental Consanguinity; number (%)		7(77.8)	4(66.7)	3(100)	-	0.26	3(75)	4(80)	0.86	
AFP (mg/dl); median (IQR)		124(65.8-468.5)	97.8(57.3-391.25)	281(103–910)	-	0.30	208.5(62.65-545.75)	124(65.8-595.5)	1	
Total B cell, cell/µL; median (IQR)		75 (56–100)	65 (54–93)	74 (67–115)	-	0.11	55 (46–61)	100 (75–120)	0.04*	
IgM memory B cells, % of total B cells; median (IQR)		3.0 (1.5–3.5)	2.0 (1.0–3.5)	3.0 (2.0–9.0)	-	0.08	1.5 (0.5–2.0)	3.5 (3.0–8.0)	0.01*	
% of switched memory B cells; median (IQR)		5.0 (4.0–10)	4.5 (2.5–5.0)	10 (7–13.5)	-	0.09	4.5 (3.0–6.2)	5.0 (4.0–11)	0.55	
IgG (mg/dl); median (IQR)		463(85-741.5)	296.5(57.5-590.25)	643(108–880)	-	0.30	85(48.5-124.5)	643(485–860)	0.01*	
IgA (mg/dl); median (IQR)		133(9.75-137.75)	133(15.25-137.75)	72.5(5.35–323.5)	-	0.86	12.5(7-104.25)	137.5(134-139.5)	0.03*	
IgM (mg/dl); median (IQR)		180(128.5–380)	186(112.75–295)	170(140–3080)	-	0.79	371(135.75-2447.5)	170(120–195)	0.22	
IgE (IU/ml); median (IQR)		1(1–5)	2.5(1-8.75)	1(1–1)	-	0.09	2.5(1-6.4)	1(1-7.25)	0.79	
CSR defect; number (%)		4(44.4)	3(50)	1(33.3)	-	0.63	-	-	-	
Immunological phenotype; number (%)	HIGM	4(44.4)	3(50)	1(33.3)**	-	4(100) - - -	4(100)	0.03	0.03*	
	Hypo gammaglobulinemia	2(22.2)	2(33.3)	-			-	2(40)		
	IgA-D	1(11.1)	-	1(33.3)			-	1(20)		
	Normal	2(22.2)	1(16.7)	1(33.3)			-	2(40)		

*Abbreviations: CSR*,* Class switching recombination; HIGM*,* hyper IgM; IgA-D*,* IgA deficiency; Ig*,* immunoglobulin; IQR*,* inter-quartile range with 75th and 25th percentiles; N*, Count. *p-value < 0.05 is statistically significant. ** This patient with mild mutation but the HIGM phenotype is 7-year-old at the time of study

The median (IQR) age of onset ataxia and telangiectasia was 1.5 (1.25-3) years and 3.5 [2–4] years, respectively. All the patients were suffering from ataxia and telangiectasia with no significant difference between their onset in different groups, and overall, other common manifestations in the A-T patients were fever (66.7%) and infection (55.6%), with a higher frequency of the respiratory infection. Lower respiratory tract infections (LRI) and recurrent/chronic upper respiratory tract infections (URI) were reported in 33.3% of the patients. Among non-infectious manifestations, hepatosplenomegaly was observed in 22.2% of A-T patients, mostly in mild phenotype. All participants were alive and without any malignancy. Detailed information on clinical manifestations and organ involvement is presented in Table 2.

**Table 2:** Clinical manifestations of patients with Ataxia-telangiectasia

Parameter	All patients (*N* = 9)	Patients with severe form (*N* = 6)	Patients with mild form (*N* = 3)	*p*. value severe vs. mild	Patients with CSR defect (*N* = 4)	Patients with CSR normal (*N* = 5)	*p*. value	
Ataxia; number (%)	9(100%)	6(100%)	3(100%)	-	4(100%)	5(100%)	-	
Telangiectasia; number (%)	9(100%)	6(100%)	3(100%)	-	4(100%)	5(100%)	-	
Fever; number (%)	6(66.7%)	4(66.7%)	2(66.7%)	1.0	4(100%)	2(40%)	0.06	
Cold; number (%)	5(55.5%)	3(50%)	2(66.7%)	0.63	2(50%)	3(60%)	0.76	
Infection; number (%)	5(55.6%)	3(50%)	2(66.7%)	0.63	3(75%)	2(40%)	0.29	
Otitis media; number (%)	1(16.7%)	1(16.7%)	0	0.45	1(25%)	0	0.24	
Recurrent/chronic URTI; number (%)	3(33.3%)	2(33.3%)	1(33.3%)	1.0	1(25%)	2(40%)	0.63	
LRTI; number (%)	3(33.3%)	2(33.3%)	1(33.3%)	0.74	3(75%)	1(20%)	0.06	
Respiratory distress; number (%)	3(33.3%)	2(33.3%)	1(33.3%)	1.0	1(25%)	2(40%)	0.63	
Thrombocytopenia; number (%)	1(11.1%)	0	1(33.3%)	0.13	1(25%)	0	0.24	
Skin manifestation; number (%)	2(22.2%)	2(33.3%)	0	0.26	2(50%)	0	0.07	
Repeated cough; number (%)	4(44.4%)	2(33.3%)	2(66.7%)	0.34	2(50%)	2(40%)	0.76	
Oral thrush; number (%)	1(11.1%)	1(16.75%)	0	0.45	0	1(20%)	0.34	
Hepatosplenomegaly; number (%)	2(22.2%)	0	2(66.7%)	0.02*	1(25%)	1(20%)	0.86	
Epilepsy; number (%)	1(11.1%)	1(16.7%)	0	0.45	0	1(20%)	0.34	
Visual impairment; number (%)	1(11.1%)	1(16.7%)	0	0.28	0	1(20%)	0.36	
Speech impairment; number (%)	4(44.4%)	2(33.3)	2(66.7%)	0.34	0	4(80%)	0.02*	
Diarrhea; number (%)	4(44.4%)	2(33.3%)	2(66.7%)	0.34	2(50%)	2(40%)	0.76	
Autoimmunity; number (%)	4(44.4%)	2(33.3%)	2(66.7%)	0.34	3(75%)	1(20%)	0.1	

*Abbreviations: LRTI*,* lower respiratory tract infection; URTI*,* upper respiratory tract infection.*

*p-value < 0.05 is statistically significant

The decreased serum Ig levels were observed in five patients (55.6%) for both IgA and IgG, whereas we recorded the increased serum levels in four patients (44.4%) for IgM in our patients (all from the CSR-D group, Table 3). In addition, rational links were observed between phenotype severity (severe and mild) and mutation severity, while 44.4% of frameshift (c.5585delA, c.3600-3601delTT, and c.6259delG) and 22.2% of nonsense (c.8907T > G, c.8050 C > T, and c.829G > T) mutations were observed in the severe form of A-T patients. However, A-T patients with mild phenotype were associated with missense (c.6452G > C, one patient) and splice-site error (c.6453–2 A > G, two patients) alternations. About 90% of A-T patients were homozygous, except for one severe form of A-T patients with compound heterozygous phenotype who had c.8907T > G and c.8050 C > T mutations (Table 4).

**Table 3:** Qualitative laboratory and immunologic data of A-T patients

Parameter	All patients (*N* = 9)	Patients with severe form (*N* = 6)	Patients with mild form (*N* = 3)	*p*. value severe vs. mild	Patients with CSR defect (*N* = 4)	Patients with CSR normal (*N* = 5)	*p*.value
AFP Frequency (%)	3(33.3%) high (20–100)	3(50%) high (20–100)	3(100%) very high (> 100)	0.13	1(25%) high (20–100)	2(40%) high (20–100)	0.63
	6(66.7%) very high (> 100)	3(50%) very high (> 100)			3(75%) very high (> 100)	3(60%) very high (> 100)	
IgG Frequency (%)	5(55.6%) decreased	4(66.7%) decreased	1(33.3%) decreased	0.34	4(100%) decreased	1(20%) decreased	0.02*
	4(44.4%) normal	2(33.3%) normal	2(66.7%) normal			4(80%) normal	
IgA Frequency (%)	5(55.6%) decreased	3(50%) decreased	2(66.7%) decreased	0.63	4(100%) decreased	1(20%) decreased	0.02*
	4(44.4%) normal	3(50%) normal	1(33.3%) normal			4(80%) normal	
IgM Frequency (%)	2(22.2%) increased	1(16.7%) increased	1(33.3%) increased	0.57	4(100%) increased	5(100%) normal	0.007*
	7(77.8%) normal	5(83.3%) normal	2(66.75) normal				

*Alpha-fetoprotein (AFP) is considered Normal < 20*,* High 20–100*,* and Very high > 100. Serum immunoglobulin values are considered via age-related normal range in references.*

**Table 4:** Genetic data of A-T patients

Parameter		Mutations	All patients (*N* = 9)	Patients with severe form (*N* = 6)	Patients with mild form (*N* = 3)	*p*. value	patients with CSR defect (*N* = 4)	patients with CSR normal (*N* = 5)	*p*. value
Mutations (%)	Nonsense	P3 (c.8907T > G, p.Y2969X + c.8050 C > T, p.Q2684X) P5 (c.829G > T, p.E277X)	P3, P5 (22.2%)	P3, P5 (33.3%)	-	0.03*	P3 (25%)	P5 (20%)	0.40
	Frameshift	P1 (c.5585delA, p.Q1862RfsX25) P4 (c.3600-3601delTT, p.F1201WfsX3) P7 (c.6259delG, p.E2087KfsX9) P8 (c.6259delG, p.E2087KfsX9)	P1, P4, P7, P8 (44.4%)	P1, P4, P8, P8 (66.7%)	-		P1, P4 (50%)	P7, P8(40%)	
	Splice error	P2 (c.6453–2 A > G) P6 (c.6453–2 A > G)	P2, P6 (22.2%)	-	P2, P6 (66.7%)		-	P2, P6 (40%)	
	Missense	P9 (c.6452G > C, p.R2151T)	P9 (11.1%)	-	P9 (33.3%)		P9(25%)	-	
Genotyping (%)			P1, P2.P4, P5, P6, P7, P8, P9 (88.9%) homozygous	P1, P4, P5, P7, P8 (83.3%) homozygous	P2, P6, P9 (100%) homozygous	0.45	P1, P4, P9 (75%) homozygous	P2, P5, P6, P7, P8 (100%) homozygous	0.24

**Fig. 1:**
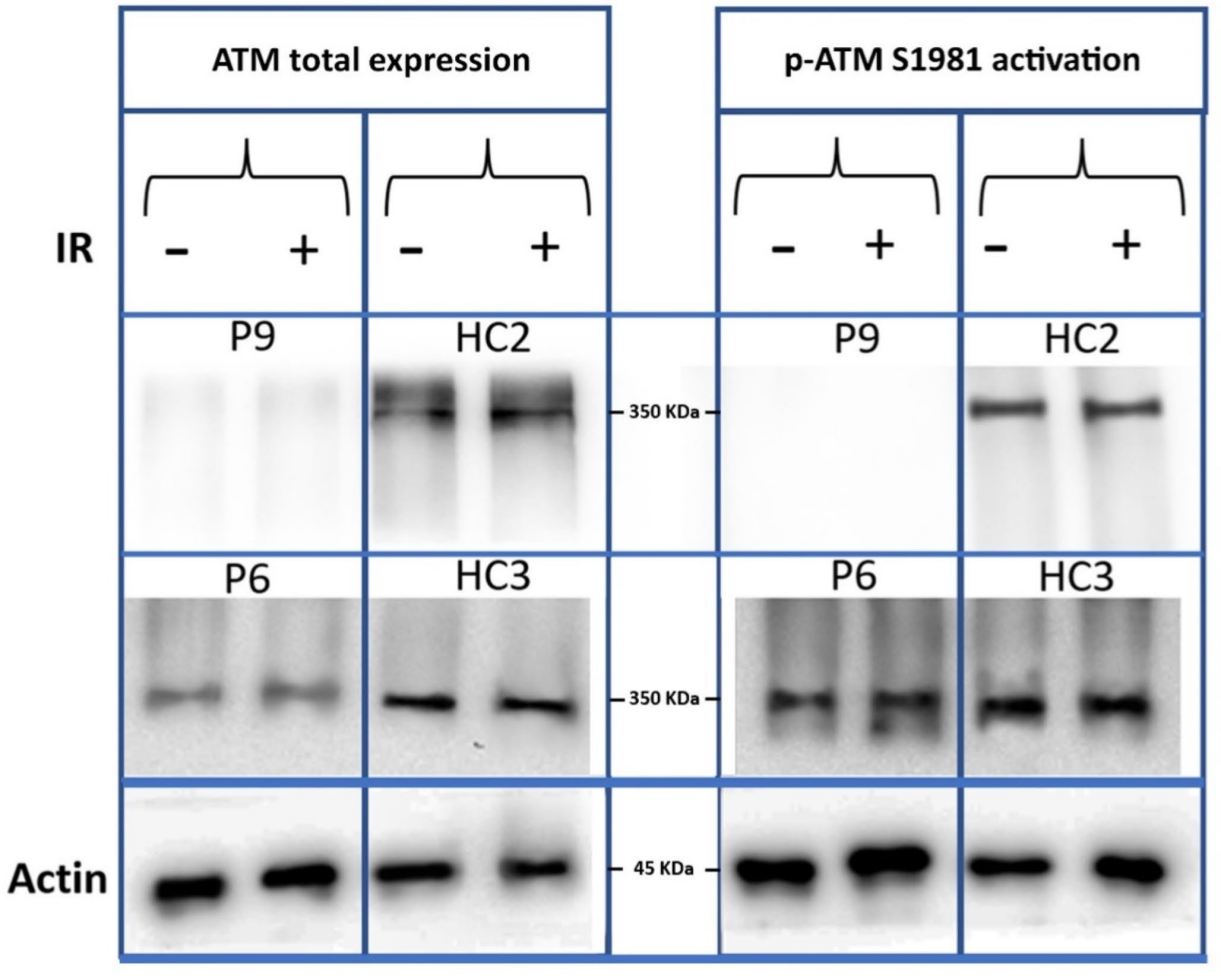
Western blot analysis of the expression and p-Ser1981- autophosphorylation of ATM protein in patient P9 (homozygous frameshift mutation of p.Glu2087LysfsTer9), P6 (homozygous splice site mutation of c.6453–2 A > G) and healthy controls (HC2 and HC3). Whole-cell extracts were collected from patients and normal ATM-positive controls and then harvested at one minute post-irradiation (0.5 Gy). Two lanes per lysate are shown, one unirradiated (-) and one irradiated (+) (activated ATM). Detectable expression and activity of ATM protein was present in P6 in comparison with HC3. P, patient; C, control; IR, irradiation

**Fig. 2:**
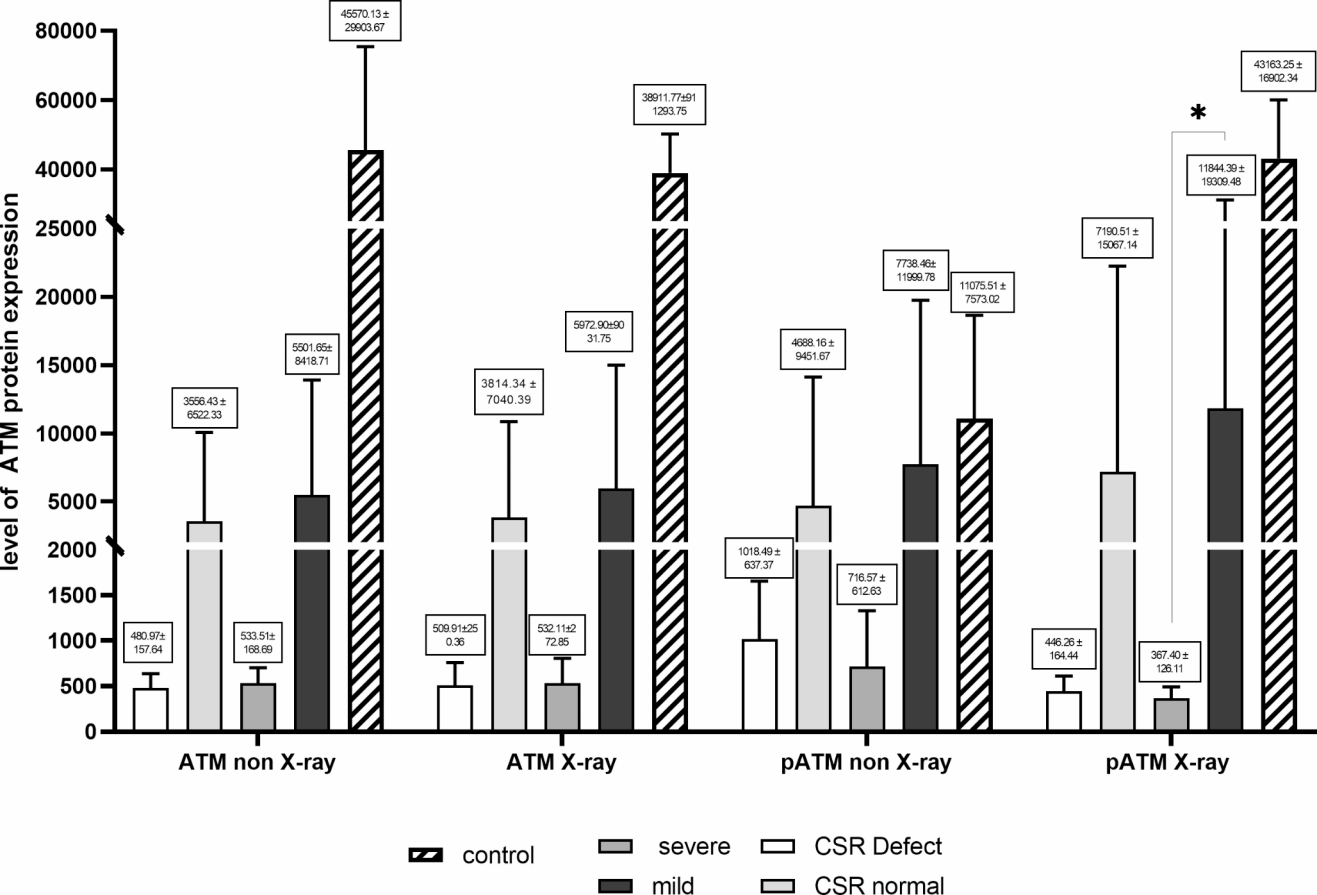
The level of phosphorylated and non-phosphorylated (p-Ser1981) ATM protein expression by western blot method in A-T patients and control group (Mean ± SD, Table S1). For more detailed presentred of data in median and interquinltie range please see Table S2. CSR: Class switching recombination. *P-value < 0.05 is statistically significant between mild and severe patients

Western blot analysis was utilized to examine the expression of ATM protein and perform p-Ser1981 autophosphorylation in patients, comparing them with healthy control individuals, as illustrated in Fig. 1. Whole-cell extracts were obtained from both patients and normal healthy controls, with harvesting taking place one minute following irradiation (0.5 Gy). Blots were probed with antibodies targeting ATM and p-ATM (p-Ser1981) to accurately assess the overall levels of these proteins within the lysate (Figs. 1 and 2). No statistically significant differences were observed between the A-T patients’ groups in all conditions, except for the comparison between severe and mild clinical phenotypes in the p-ATM after X-ray stimulation. It is worth noting that in all experiments, the expression of ATM and p-ATM was comparably higher in healthy controls (*p* < 0.05). Moreover, patients with mild clinical phenotype and normal CSR had the highest expression of ATM independent of X-ray stimulation. It becomes evident that ATM expression in severe patients decreased by about 23% after irradiation, while ATM expression increased by around 60% after irradiation. However, the pattern of ATM activity was slightly different (Table S1). Patients with mild presentations still showed the top p-ATM level, but cases with CSR-D had more activated p-ATM before irradiation, while after stimulation, the p-ATM level decreased subsequently to the level of CSR normal cases. Of note, mean p-ATM expression in severe cases 48% decreased after irradiation while mean p-ATM levels increased 53% for mild patients. In a similar fashion, mean p-ATM expression in CSR-D cases was reduced after irradiation (-56%), while mean p-ATM expression in CSR-N patients seemed to increase slightly (+ 53%). (Fig. 2, Table S1).

## Discussion

Ataxia telangiectasia is characterized by a heightened sensitivity to radiation and inborn errors of immunity, both of which have been evaluated by in-vivo and in-vitro assays in A-T patients. To gain a better understanding of the mechanism of this sensitivity, it is crucial to identify the molecular defect related to ATM protein activation. This should be initiated by the evaluation of residual expression or function in affected individuals and be linked with the prognosis of the disease. In our study, we investigated nine A-T patients with an assessment of their ATM expression and activation as the p-ATM under radiation.

The median age of diagnosis of A-T in our patients was 3 (IQR: 1.75–7.5) years, which was lower than that reported in other studies: 11 (IQR: 5.7–17) years [30], 5 (IQR:3–7) years [25], and 5.3 (IQR: 2.9-8.0) years [31]. Patients with A-T are rarely diagnosed in their first year of life, as their typical neurological manifestations are noted at a later age, often leading to misdiagnosis. Early detection in newborns can lead to a correct diagnosis and early interventions, including specialized care for pulmonary function, prophylactic antibiotics or Ig replacement therapy for recurrent infections, and adapted treatment for malignancies. Classic A-T patients have no symptoms in their first year of life, but progressive symptoms start shortly thereafter. Therefore, an early diagnosis and predictor of the severity of the disease are crucial in providing appropriate medical support and interventions for these patients.

We observed that IgA and IgG serum levels decreased in the five A-T patients. A-T is characterized by deficiencies in both humoral and cellular immunity, as evidenced by frequent reports of class-switched Ig defects and impaired antibody response to various antigens [32, 33]. A recent report [32] noted the predictive role of IgA as a simple surrogate marker in anticipating the poorer prognosis in A-T patients. Studies have shown that abnormalities in Ig profiles and defective polysaccharide antibody synthesis are common in A-T [32–36]. Research has also demonstrated that when the DNA damage response pathway is deactivated, as is the case in A-T B-cells, there is a hindrance in the process of CSR of IgM toward IgG and IgA [21, 37]. Impaired double-strand break repair processes such as V(D)J recombination and CSR, which are characterized by a low number of circulating T-cells and B-cells, may also be involved in low or absent serum IgA and IgG2. An estimated 10% of A-T patients have low IgA and IgG levels with normal to high IgM levels, which is known as the ‘hyper IgM phenotype with hypogammaglobulinemia’ (AT-HIGM) [36]. The residual function of ATM proteins specific for Ig CSR may be related to their general presentation and capacity for recognition and response to DNA DSB [38].

Insufficient production of class-switched antibodies corresponds to a higher risk of respiratory tract infections, including pneumonia [39], which is also observed in our patients. According to divided patient groups, patients with a severe phenotype of the disease mostly had severe genetic mutations, including frameshift mutations, while in patients who were regarded as in the mild group, two cases had splice errors, and one case had a missense mutation. These observations confirm that the severity of the clinical phenotype is influenced at least partially by the ATM genotype. Understanding the type of mutation that causes A-T can provide knowledge about the disease’s molecular and pathophysiological foundations [3]. In other words, many frameshift or nonsense mutations in *ATM* result in a truncation of the ATM protein. Individuals with these types of mutations possibly exhibit a severe phenotype. Additionally, missense or splice-site mutations result in a milder form of the disease caused by residual ATM kinase activity [40, 41]. Inactive homodimeric ATM dissociates into active monomers for autophosphorylation after DSB formation. The MRE11-RAD50-NBS1 (MRN) complex then recruits activated ATMs to DSBs in order to rapidly phosphorylate downstream effectors, thereby facilitating DNA repair and maintaining genomic integrity [42, 43]. While establishing a definitive correlation between CSR-D or CSR-N and clinical severity in A-T patients is complex due to the broad spectrum of clinical manifestations, the variability in clinical and immunological features suggests the potential influence of genetic modifiers [26]. Furthermore, immunological studies have demonstrated substantial variability in B and T cell subsets among A-T patients, highlighting the diverse immunological landscape within this genetic disorder. The extent of an individual’s T and B cell functional defects which is not restricted only to CSR-D likely contributes to the observed heterogeneity in clinical presentations [30].

ATM complete inhibition has been shown to make tumor cells more vulnerable to DNA-damaging therapies [44]. Furthermore, primary cells derived from A-T patients and ATM-knockout mice with severe mutations were more sensitive to DSB-inducing radiation or chemotherapy reagents [45]. While patients P5, P7, and P8 exhibited normal CSR activity despite severe genotypes suggesting absent ATM expression, the potential compensatory role of ATM- and Rad3-related (ATR) kinase remains unclear. Although ATM, a master DNA damage response (DDR) kinase, is critical for CSR by suppressing S region DSB resection, ATR, another related DDR kinase, plays a similar role. Recent data suggests ATR promotes CSR by regulating cell proliferation upon damage without negatively impacting DSB end-joining features [46].

In this study, we set up 0.5 Gy X-ray radiation to stimulate ATM protein to phosphorylation (p-ATM). DSBs formed in cells at doses ranging from 1 mGy to 0.5 Gy; however, the extent of DSB formation varied depending on the cell species [47]. Ionizing radiation-inducible kinase activity has been discovered in p-ATM. Cells from patients with A-T have severely impaired cell cycle checkpoints in response to gamma radiation. p-ATM has been suggested to be a component of a complex that detects DNA damage, specifically DSBs [48]. We investigated how p-ATM expression changes in response to 0.5 Gy radiation in PBMCs from cases with mutated *ATMs* and healthy controls. It should be emphasized that our current observation showed that radiation increased the p-ATM responses in healthy controls without significant change in the total protein expression of ATM, however in ATM-deficient patients only increased level of p-ATM responses can be evident marginally in mild mutation and cases with normal CSR capacity. Expectedly the level of p-ATM is not changed in severe clinical cases and CSR-D patients since they have no or very little expression of protein in general.

The level of both ATM and p-ATM protein expression was significantly lower in all patients than in the healthy control group, both before and after X-ray radiation. In these patients, low ATM protein expression and phosphorylation activity are due to the presence of mutations or probably amino acid deletions, which may lead to early nonsense-mediated mRNA decay and non-efficient post-translational regulation. Moreover, ATM and p-ATM protein expression in the mild A-T phenotype was higher than in severe forms of the disease, both before and after X-ray radiation. Although there were no statistically significant variances detected among and within the groups under static conditions, it is important to highlight that, across all experiments, the levels of ATM and p-ATM expression in mild phenotype were consistently higher compared to other patients, but this level was not correlated with the CSR defects in AT-patients. Perhaps a positive increase in ATM phosphorylation after irradiation is sufficient to rescue CSR or clinical severity. This might be improved with the inclusion of more AT patients from other centers in future studies as the limited number of patients in this study impacted the power of statistical analysis.

## Conclusion

In summary, this study suggests that ATM and p-ATM protein expression may have clinical implications for prognostic evaluation and the severity of ataxia telangiectasia symptoms. While the findings offer a better understanding of the potential impact of X-ray radiation on ATM expression, further research is needed to fully elucidate the relationship between protein expression levels and the exact effect of radiation dose in A-T patients’ cells.

## Electronic Supplementary Material

Below is the link to the electronic supplementary material.

Supplementary Material 1

## Data Availability

No datasets were generated or analysed during the current study.

## References

[1] Van Os NJH, Chessa L, Weemaes CMR, Van Deuren M, Fiévet A, Van Gaalen J et al. Genotype-phenotype correlations in ataxia telangiectasia patients with ATM c.3576G > A and c.8147T > C mutations. J Med Genet. 2019. 10.1136/jmedgenet-2018-105635 30819809

[2] Zaki-Dizaji M, Akrami SM, Abolhassani H, Rezaei N, Aghamohammadi A. Ataxia telangiectasia syndrome: moonlighting ATM. Expert Rev Clin Immunol. 2017;13(12):1155–72. 29034753 10.1080/1744666X.2017.1392856

[3] Lavin MF, Shiloh Y. The genetic defect in Ataxia-Telangiectasia. Annu Rev Immunol. 1997;15(1):177–202. 9143686 10.1146/annurev.immunol.15.1.177

[4] Rothblum-Oviatt C, Wright J, Lefton-Greif MA, McGrath-Morrow SA, Crawford TO, Lederman HM. Ataxia telangiectasia: a review. Orphanet J Rare Dis. 2016. 10.1186/s13023-016-0543-7 PMC5123280 27884168

[5] Fattahi Z, Beheshtian M, Mohseni M, Poustchi H, Sellars E, Nezhadi SH et al. Iranome: a catalog of genomic variations in the Iranian population. Hum Mutat. 2019. 10.1002/humu.23880 31343797

[6] Cooper TJ, Wardell K, Garcia V, Neale MJ. Homeostatic regulation of meiotic DSB formation by ATM/ATR. Exp Cell Res. 2014. 10.1016/j.yexcr.2014.07.016 25116420

[7] Weitering TJ, Takada S, Weemaes CMR, van Schouwenburg PA, van der Burg M. ATM: translating the DNA damage response to adaptive immunity. Trends in Immunology; 2021. 10.1016/j.it.2021.02.001 33663955

[8] Wang Q, Chen Y, Chang H, Hu T, Wang J, Xie Y et al. The role and mechanism of ATM-Mediated autophagy in the Transition from Hyper-Radiosensitivity to Induced Radioresistance in Lung Cancer under Low-Dose Radiation. Front Cell Dev Biol. 2021. 10.3389/fcell.2021.650819 PMC8149741 34055781

[9] Chaudhary MW, Al-Baradie RS. Ataxia-telangiectasia: future prospects. Application Clin Genet. 2014. 10.2147/TACG.S35759 PMC4173637 25258552

[10] Karimian A, Ahmadi Y, Yousefi B. Multiple functions of p21 in cell cycle, apoptosis and transcriptional regulation after DNA damage. DNA Repair. 2016. 10.1016/j.dnarep.2016.04.008 27156098

[11] Samara A, Gusman M, Aker L, Parsons MS, Mian AY, Eldaya RW. The Forgotten Phacomatoses: a neuroimaging review of Rare Neurocutaneous disorders. Curr Probl Diagn Radiol. 2022. 10.1067/j.cpradiol.2021.07.002 34607749

[12] Zaki-Dizaji M, Akrami SM, Azizi G, Abolhassani H, Aghamohammadi A. Inflammation, a significant player of Ataxia–telangiectasia pathogenesis? Inflamm Res. 2018;67(7):559–70. 29582093 10.1007/s00011-018-1142-y

[13] Perlman S, Becker-Catania S, Gatti RA. Ataxia-telangiectasia: diagnosis and treatment. Semin Pediatr Neurol; 2003. 10.1016/s1071-9091(03)00026-3 14653405

[14] Satitsuksanoa P, Daanje M, Akdis M, Boyd SD, van de Veen W. Biology and dynamics of B cells in the context of IgE-mediated food allergy. Allergy: Eur J Allergy Clin Immunol. 2021. 10.1111/all.14684 33274454

[15] Mohammadinejad P, Abolhassani H, Aghamohammadi A, Pourhamdi S, Ghosh S, Sadeghi B, et al. Class switch recombination process in Ataxia Telangiectasia patients with elevated serum levels of IgM. J Immunoass Immunochem. 2015;36(1):16–26. 10.1080/15321819.2014.891525 24568663

[16] Banerji J, Olson L, Schaffner W. A lymphocyte-specific cellular enhancer is located downstream of the joining region in immunoglobulin heavy chain genes. Cell. 1983. 10.1016/0092-8674(83)90015-6 6409418

[17] Lavin MF, Gueven N, Bottle S, Gatti RA. Current and potential therapeutic strategies for the treatment of ataxia-telangiectasia. Br Med Bull. 2007;81–82(1):129–47. 17586848 10.1093/bmb/ldm012

[18] Ottaviano G, Georgiadis C, Gkazi SA, Syed F, Zhan H, Etuk A et al. Phase 1 clinical trial of CRISPR-engineered CAR19 universal T cells for treatment of children with refractory B cell leukemia. Sci Transl Med. 2022;14(668). 10.1126/scitranslmed.abq3010 36288281

[19] Witzel S, Maier A, Steinbach R, Grosskreutz J, Koch JC, Sarikidi A, et al. Safety and Effectiveness of Long-Term Intravenous Administration of Edaravone for treatment of patients with amyotrophic lateral sclerosis. JAMA Neurol. 2022;79(2):121. 35006266 10.1001/jamaneurol.2021.4893 PMC8749709

[20] Cortese I, Muranski P, Enose-Akahata Y, Ha SK, Smith B, Monaco M, et al. Pembrolizumab Treatment for Progressive Multifocal Leukoencephalopathy. N Engl J Med. 2019;380(17):1597–605. 30969503 10.1056/NEJMoa1815039

[21] Amirifar P, Mozdarani H, Yazdani R, Kiaei F, Moeini Shad T, Shahkarami S, et al. Effect of class switch recombination defect on the phenotype of Ataxia-telangiectasia patients. Immunol Invest. 2021;50(2–3):201–15. 32116070 10.1080/08820139.2020.1723104

[22] Abolhassani H, Kiaee F, Tavakol M, Chavoshzadeh Z, Mahdaviani SA, Momen T et al. Fourth update on the Iranian National Registry of primary immunodeficiencies: integration of molecular diagnosis. J Clin Immunol. 2018. 10.1007/s10875-018-0556-1 30302726

[23] Seidel MG, Kindle G, Gathmann B, Quinti I, Buckland M, van Montfrans J et al. The European Society for Immunodeficiencies (ESID) Registry Working definitions for the clinical diagnosis of inborn errors of immunity. J Allergy Clin Immunology: Pract. 2019. 10.1016/j.jaip.2019.02.004 30776527

[24] Abolhassani H, Chou J, Bainter W, Platt CD, Tavassoli M, Momen T, et al. Clinical, immunologic, and genetic spectrum of 696 patients with combined immunodeficiency. J Allergy Clin Immunol. 2018;141(4):1450–8. 28916186 10.1016/j.jaci.2017.06.049

[25] Amirifar P, Ranjouri MR, Pashangzadeh S, Lavin M, Yazdani R, Moeini Shad T, et al. The spectrum of ATM gene mutations in Iranian patients with ataxia-telangiectasia. Pediatr Allergy Immunol. 2021;32(6):1316–26. 33547824 10.1111/pai.13461

[26] Amirifar P, Mehrmohamadi M, Ranjouri MR, Akrami SM, Rezaei N, Saberi A et al. Genetic risk variants for Class switching recombination defects in Ataxia-Telangiectasia patients. J Clin Immunol. 2022. 10.1007/s10875-021-01147-8 PMC8821084 34628594

[27] Aghamohammadi A, Rezaei N, Yazdani R, Delavari S, Kutukculer N, Topyildiz E et al. Consensus Middle East and North Africa Registry on Inborn errors of immunity. J Clin Immunol. 2021. 10.1007/s10875-021-01053-z PMC8310844 34052995

[28] Reiman A, Srinivasan V, Barone G, Last JI, Wootton LL, Davies EG et al. Lymphoid tumours and breast cancer in ataxia telangiectasia; substantial protective effect of residual ATM kinase activity against childhood tumours. Br J Cancer. 2011. 10.1038/bjc.2011.266 PMC3170966 21792198

[29] Dörk T, Bendix-Waltes R, Wegner RD, Stumm M. Slow progression of Ataxia-Telangiectasia with double missense and in Frame splice mutations. Am J Med Genet. 2004. 10.1002/ajmg.a.20601 15054841

[30] Moeini Shad T, Yazdani R, Amirifar P, Delavari S, Heidarzadeh Arani M, Mahdaviani SA et al. Atypical Ataxia Presentation in Variant Ataxia Telangiectasia: Iranian Case-Series and Review of the Literature. Vol. 12, Frontiers in immunology. Switzerland; 2021. p. 779502. 10.3389/fimmu.2021.779502 PMC8795590 35095854

[31] Micol R, Slama L, Ben, Suarez F, Le Mignot L, Beauté J, Mahlaoui N, et al. Morbidity and mortality from ataxia-telangiectasia are associated with ATM genotype. J Allergy Clin Immunol. 2011;128(2):382–9. 21665257 10.1016/j.jaci.2011.03.052

[32] Zielen S, Duecker RP, Woelke S, Donath H, Bakhtiar S, Buecker A, et al. Simple measurement of IgA predicts immunity and mortality in Ataxia-Telangiectasia. J Clin Immunol. 2021;41(8):1878–92. 34477998 10.1007/s10875-021-01090-8 PMC8604875

[33] Reichenbach J, Schubert R, Feinberg J, Beck O, Rosewich M, Rose MA, et al. Impaired interferon-γ production in response to live bacteria and toll-like receptor agonists in patients with ataxia telangiectasia. Clin Exp Immunol. 2006;146(3):381–9. 17100756 10.1111/j.1365-2249.2006.03221.x PMC1810411

[34] Aghamohammadi A, Imai K, Moazzami K, Abolhassani H, Tabatabaeiyan M, Parvaneh N et al. Ataxia-Telangiectasia in a Patient Presenting With Hyper-immunoglobulin M Syndrome A Aghamohammadi. Vol. 20, J Investig Allergol Clin Immunol. 2010. Available from: www.ncbi.nlm.nih.gov/snp 20945614

[35] Ghiasy S, Parvaneh L, Azizi G, Sadri G, Zaki dizaji M, Abolhassani H, et al. The clinical significance of complete class switching defect in Ataxia telangiectasia patients. Expert Rev Clin Immunol. 2017;13(5):499–505. 28162005 10.1080/1744666X.2017.1292131

[36] van Os NJH, Jansen AFM, van Deuren M, Haraldsson A, van Driel NTM, Etzioni A, et al. Ataxia-telangiectasia: Immunodeficiency and survival. Clin Immunol. 2017;178:45–55. 28126470 10.1016/j.clim.2017.01.009

[37] Panchakshari RA, Zhang X, Kumar V, Du Z, Wei PC, Kao J, et al. DNA double-strand break response factors influence end-joining features of IgH class switch and general translocation junctions. Proc Natl Acad Sci U S A. 2018;115(4):762–7. 29311308 10.1073/pnas.1719988115 PMC5789958

[38] Lumsden JM, McCarty T, Petiniot LK, Shen R, Barlow C, Wynn TA, et al. Immunoglobulin class switch recombination is impaired in atm-deficient mice. J Exp Med. 2004;200(9):1111–21. 15504820 10.1084/jem.20041074 PMC2211853

[39] Demirdag YY, Gupta S. Update on infections in primary antibody deficiencies. Front Immunol. 2021;12:634181. 33643318 10.3389/fimmu.2021.634181 PMC7905085

[40] Verhagen MMM, Last JI, Hogervorst FBL, Smeets DFCM, Roeleveld N, Verheijen F, et al. Presence of ATM protein and residual kinase activity correlates with the phenotype in ataxia-telangiectasia: a genotype-phenotype study. Hum Mutat. 2012;33(3):561–71. 22213089 10.1002/humu.22016

[41] Taylor P, Li Y, Camp S, Rachinsky TL, Ekström T, Getman D, et al. Structure and regulation of expression of the acetylcholinesterase gene. Chem Biol Interact. 1993;87(1–3):199–207. 8343976 10.1016/0009-2797(93)90043-x

[42] Lee JH, Paull TT. Activation and regulation of ATM kinase activity in response to DNA double-strand breaks. Oncogene. 2007;26(56):7741–8. 18066086 10.1038/sj.onc.1210872

[43] Lavin MF. ATM and the Mre11 complex combine to recognize and signal DNA double-strand breaks. Oncogene. 2007;26(56):7749–58. 18066087 10.1038/sj.onc.1210880

[44] Jiang H, Reinhardt HC, Bartkova J, Tommiska J, Blomqvist C, Nevanlinna H, et al. The combined status of ATM and p53 link tumor development with therapeutic response. Genes Dev. 2009;23(16):1895–909. 19608766 10.1101/gad.1815309 PMC2725944

[45] Lee HJ, Lan L, Peng G, Chang WC, Hsu MC, Wang YN, et al. Tyrosine 370 phosphorylation of ATM positively regulates DNA damage response. Cell Res. 2015;25(2):225–36. 25601159 10.1038/cr.2015.8 PMC4650576

[46] Sun X, Liu M, Bai J, Xu J, Zhu C, Dong J et al. ATR kinase activity promotes antibody class switch recombination in B cells through cell cycle regulation without suppressing DSB resection and microhomology usage. J Leukoc Biol. 2021. 10.1002/JLB.2MA0321-064R 33884660

[47] Shimura N, Kojima S. The Lowest Radiation Dose having molecular changes in the living body. Dose Response. 2018;16(2):1559325818777326. 29977175 10.1177/1559325818777326 PMC6024299

[48] Bruine de Bruin L, Schuuring E, de Bock GH, Slagter-Menkema L, Mastik MF, Noordhuis MG, et al. High pATM is Associated with Poor Local Control in Supraglottic Cancer treated with Radiotherapy. Laryngoscope. 2020;130(8):1954–60. 32275333 10.1002/lary.28641 PMC7384019

