# Supplementary material for: ATM Expression and Activation in Ataxia Telangiectasia Patients with and without Class Switch Recombination Defects
Source: J Clin Immunol. 2025 Jan 24;45(1):67. doi: 10.1007/s10875-025-01857-3 (PMC11762072; doi:10.1007/s10875-025-01857-3)
Supplement: Supplementary file 1 — Supplementary Material 1 [file 10875_2025_1857_MOESM1_ESM.docx]

**Supplementary Data**

**ATM Expression and Activation in Ataxia Telangiectasia Patients with and without Class Switch Recombination Defects**

**Table S1: Comparison of phosphorylated and non-phosphorylated (p-Ser1981) ATM protein expression between the patients and controls**

| Items (number) | **ATM - non-X-ray**  **(Mean ± SD)** | **ATM - X-ray**  **(Mean ± SD)** | **P value** | **phosphorylated ATM- non-X-ray (Mean ± SD)** | **phosphorylated ATM- X-ray**  **(Mean ± SD)** | **P value** |
| --- | --- | --- | --- | --- | --- | --- |
| **All patients (9)** | 2189.55 ± 4889.48 | 2345.70 ± 5276.38 | 0.51 | 3057.19 ± 6968.50 | 4193.06 ± 11231.84 | 0.31 |
| **Control (3)** | 45570.13 ± 29903.67 | 38911.77 ± 911293.75 | 1.0 | 11075.51 ± 7573.02 | 43163.25 ± 16902.34 | 0.1 |
| **P value** | 0.01** | 0.01** | - | 0.05 | 0.03** | - |
| patients with severe form (6) | 533.51± 168.69 | 532.11 ± 272.85 | 0.91 | 716.57 ± 612.63 | 367.40 ± 126.11 | 0.6 |
| patients with mild form (3) | 5501.65 ± 8418.71 | 5972.90 ± 9031.75 | 0.28 | 7738.46 ± 11999.78 | 11844.39 ± 19309.48 | 0.28 |
| **P value** | 0.12 | 0.07 | - | 0.07 | 0.02* | - |
| patients with CSR defect (4) | 480.97± 157.64 | 509.91±250.36 | 1.0 | 1018.49 ± 637.37 | 446.26 ± 164.44 | 0.06 |
| patients with CSR normal (5) | 3556.43 ± 6522.33 | 3814.34 ± 7040.39 | 0.68 | 4688.16 ± 9451.67 | 7190.51 ± 15067.14 | 0.50 |
| **P value** | 0.08 | 0.22 | - | 0.46 | 0.62 | - |
| **CSR: Class switching recombination. *P-value < .05 is statistically significant between mild and severe patients highlighted in Figure 2.**  ****P-value < .05 is statistically significant between patients and controls.** | | | | | | |

**Table S2: The levels of phosphorylated and non-phosphorylated (p-Ser1981) ATM protein expression in A-T patients and controls based on IQR.**

| **Items (number)** | **ATM - non-X-ray (25th - 75th percentiles)** | **ATM - X-ray (25th - 75th percentiles)** | **phosphorylated ATM- non-X-ray (25th - 75th percentiles)** | **phosphorylated ATM- X-ray (25th - 75th percentiles)** |  |
| --- | --- | --- | --- | --- | --- |
| **All patients (9)** | 528.4(474.2-775.3) | 628.46(383.0-917.9) | 606.9(471.2-1453.4) | 421.4(305.2-696.1) |  |
| **Control (3)** | 31724.6(25098.3-79887.3) | 45260.2(25872.3-45602.7) | 15067.6(2341.66-15817.2) | 33999.5(32821.6-62668.5) |  |
| patients with severe form (6) | 505.0(421.6-694.7) | 464.58(308.6-728.8) | 568.8(346.5-938.5) | 360.4(263.3-459.0) |  |
| patients with mild form (3) | 782.6(500.9-15221.3) | 806.06(710.9-16401.7) | 973.4(648.6-21593.3) | 789.8(602.5-34140.8) |  |
| patients with CSR defect (4) | 483.9(331.0-627.9) | 471.5(296.4-761.6) | 790.1(571.8-1693.4) | 450.4(293.4-594.9) |  |
| patients with CSR normal (5) | 768.1(505.0-8002.0) | 710.9(464.5-8715.8) | 577.5(310.6-11120.9) | 421.4(300.2-17465.3) |  |
| **CSR: Class switching recombination.** | | | | | |

**Table S3: Molecular, clinical severity, CSR classification, and effect of X-ray of ATM protein expression in A-T patients.**

| **Patients** | **Mutation** | **Severe/mild** | **CSR-D/CSR-N** | **ATM - non-X-ray** | **ATM - X-ray** | **phosphorylated ATM- non-X-ray** | **phosphorylated ATM- X-ray** |
| --- | --- | --- | --- | --- | --- | --- | --- |
| **P1** | c.5585delA, p.Q1862RfsX25 | severe | CSR-D | 285.76 | 628.46 | 1933.48 | 572.01 |
| **P2** | c.6453-2A>G | mild | CSR-N | 782.62 | 710.91 | 648.62 | 789.84 |
| **P3** | c.8907T>G, p.Y2969X +  c.8050C>T, p.Q2684X | severe | CSR-D | 466.89 | 290.43 | 560.14 | 281.65 |
| **P4** | c.3600-3601delTT, p.F1201WfsX3 | severe | CSR-D | 670.3 | 314.69 | 606.93 | 328.83 |
| **P5** | c.829G>T, p.E277X | severe | CSR-N | 768.11 | 477.8 | 577.55 | 392.14 |
| **P6** | c.6453-2A>G | mild | CSR-N | 15221.38 | 16401.72 | 21593.35 | 34140.8 |
| **P7** | c.6259delG, p.E2087KfsX9 | severe | CSR-N | 481.6 | 451.37 | 238.85 | 208.38 |
| **P8** | c.6259delG, p.E2087KfsX9 | severe | CSR-N | 528.42 | 1029.91 | 382.44 | 421.41 |
| **P9** | c.6452G>C, p.R2151T | mild | CSR-D | 500.94 | 806.06 | 973.42 | 602.53 |
| **CSR-D: defects in class switching recombination; CSR-N: Normal class switching recombination** | | | | | | | |
